# Exploring Breed-Specific Milk Coagulation in Spanish Dairy Sheep: A Canonical Correlation Approach

**DOI:** 10.3390/ani14060900

**Published:** 2024-03-14

**Authors:** Javier Caballero-Villalobos, Ana Garzón, Elena Angón, Ramón Arias, Alessio Cecchinato, Nicolò Amalfitano, José M. Perea

**Affiliations:** 1 Departamento de Producción Animal, Universidad de Córdoba, 14071 Córdoba, Spain; pa1gasia@uco.es (A.G.); eangon@uco.es (E.A.); jmperea@uco.es (J.M.P.); 2 Centro Regional de Selección y Reproducción Animal de Castilla-La Mancha, 13300 Valdepeñas, Spain; rarias@jccm.es; 3 Department of Agronomy, Food, Natural Resources, Animals and Environment (DAFNAE), University of Padova, Viale dell’Università 16, 35020 Padova, Italy; alessio.cecchinato@unipd.it (A.C.); nicolo.amalfitano@unipd.it (N.A.)

**Keywords:** sheep milk, coagulation, breed, canonical correlation, Manchega, Assaf, Merino

## Abstract

**Simple Summary:**

The transformation of milk into cheese is a complex process influenced by various factors, including the breed of the sheep providing the milk. Our study focused on understanding how different Spanish dairy sheep breeds affect the quality and properties of milk intended for cheesemaking. We examined milk samples from four different breeds, analyzing their composition and how they coagulate, a key step in cheese production. We found that while there are some common patterns in how milk from these breeds coagulates, certain breeds have unique characteristics. Our research highlights the importance of considering breed selection in coagulation, offering insights that could lead to better cheese production techniques.

**Abstract:**

The transformation of milk into cheese largely depends on the technological properties of the raw material, with breed being a crucial factor that influences both the composition and coagulation properties of the milk used for cheesemaking. This study uses canonical correlation analysis to explore the relationships between physicochemical traits and coagulation properties in milk from various Spanish breeds, aiming to identify both common and breed-specific patterns that impact milk technological aptitude. A total of 832 milk samples from Manchega, Assaf, Merino de Grazalema, and Merino de Los Pedroches breeds were analyzed. The milk characteristics investigated included pH, composition (fat, protein, lactose, total solids), and coagulation properties (curd firmness—A_60_, rennet clotting time—RCT, curd firming time—k_20_, and individual laboratory curd yield—ILCY). The results reveal a shared correlation structure across breeds and unique covariation patterns in some breeds that deviate from the general trend. While Assaf and Merino de Los Pedroches follow the common correlation pattern, Manchega and Merino de Grazalema exhibit distinct patterns. This research underscores the need for in-depth study and suggests that the dairy industry could benefit from shifting from the traditional focus on maximizing fat and protein for higher curd yields to considering technological traits for selective breeding.

## 1. Introduction

Dairy sheep production receives special attention in the European Union, since it is linked to less favoured regions with few alternatives for economic activity [1]. The existence of these systems potentially allows the protection of natural resources, the preservation of lifestyles, and the prevention of rural abandonment [2]. However, in recent decades, socioeconomic and structural limitations have arisen as important challenges for the sector, highlighting the vulnerability of dairy sheep production and the need to provide added value to dairy products [3,4].

Sheep milk is rarely consumed in its liquid form, being almost entirely used for cheesemaking purposes [5,6]. This situation intensifies in Mediterranean countries, where the climate and environmental conditions are optimal for the production of a great variety of unique and distinctive cheeses with a strong sociocultural heritage [7,8], often covered by quality labels such as protected designation of origin (PDO) and protected geographical indication (PGI) [9,10]. Ewe milk is known to be rich in major components, particularly in fat, protein, and total solids [11], making it more suitable for its transformation into cheese than milk from other domestic species [12,13]. The quality of this raw material is therefore not only defined as its aptitude for human consumption, but also as its suitability for processing [14] where potential degradation can critically impact farmers’ profitability [15]. 

Currently, most dairy sheep breeding programs are based on selection by milk yield only, while, in only a few cases, fat and protein are also included as selection criteria [16,17,18,19]. However, many authors have emphasised the convenience of including milk coagulation properties in these schemes [20,21] since transformation of the raw material into cheese largely relies on the milk’s technological performance [22]. Within the same species it is well known that milk traits can vary greatly, and several studies have highlighted breed as one of the most important factors that impact milk quality [23,24]. Dairy sheep systems in the Mediterranean basin involve a wide range of autochthonous breeds with differences in production due to their own particular environments and farming systems [25]. This acquires great importance in this geographical area, since it is acknowledged that even small variations can largely impact the suitability of raw milk for the manufacture of dairy products [26].

Spain holds the largest population of sheep in the European Union, representing 24.5% of the EU and 1.1% of the world sheep population [27]. This significant population is mainly spread across several different regions of the Iberian Peninsula, where a large number of autochthonous dairy breeds are raised in extensive or semi-extensive systems, alongside a few other more productive foreign breeds seeking some increase in the level of intensification [28,29]. Therefore, the aim of this study was to examine the relationships between physicochemical traits and the results of rennet coagulation evaluation on milk samples from different dairy sheep breeds used in Spain, in order to identify quality traits that determine the milk’s technological performance and curd yield. In addition, the present study also aims to explore if relationships between these traits are common to the analyzed breeds or if, to the contrary, they are breed-specific.

## 2. Materials and Methods

### 2.1. Animals, Breeds, and Husbandry Systems

The present study involves the following breeds described in the Official Catalogue of Spanish Livestock Breeds [30]: Manchega (a local enhancement breed), Assaf (a third country breed), and two varieties of Spanish Merino (Merino de Grazalema—an endangered native breed, and Merino de Los Pedroches—a Merino population nucleus oriented in recent years towards milk production). 

Manchega is the autochthonous dairy sheep breed with the largest population in Spain, with approximately 500,000 ewes in 538 flocks [31]. It is predominantly distributed in the region of Castilla-La Mancha and is utilized for producing the raw material for the PDO cheese ‘Manchego’ [32]. The farming system is semi-extensive, traditionally associated with grazing natural pastures, residues, and the remains of cereal crops [33]. It is known for its high adaptability to dry climates, ease of lambing, high longevity, and strong maternal instinct, with an average production of 232 kg in 150 days [31].

Assaf is an integrated breed that has gradually spread mainly across the Castilla y León region since its introduction into Spain in the 1980s, and is often used for crossbreeding with Churra and Castellana ewes. Currently, the population consists of approximately 130,000 ewes in 112 flocks [34]. The production system is intensive, with long lactations of ~400 kg in 180 days [35].

Merino de Grazalema is a Spanish variety of Merino sheep currently considered an independent endangered breed, consisting of approximately 5000 ewes in 33 farms dedicated to milk production for the elaboration of artisanal cheeses, recognized for their differentiated quality [34]. The population is distributed in Sierra de Grazalema Natural Park (Cádiz), where sheep are raised under extensive or semi-extensive systems, mainly based on grazing natural pastures in mountainous areas [36]. This breed is particularly adapted to cold and humidity, with average lactations of 120.22 kg of milk in 156 days [34].

Merino de Los Pedroches represents a significant group of Merino sheep from the region Valle de Los Pedroches (Córdoba) [37,38]. While it has not yet received official classification as an independent breed, the latest advancements in selection for dairy production have rendered it a noteworthy subject for comparison with the rest of breeds in this study. The production system is semi-extensive, relying on the grazing of natural forages and supplementary feeding during lactation. Milk yields are generally low (30 to 50 L per lactation), and all the production is used for the elaboration of both hard-pressed paste or torta-type cheeses [39].

### 2.2. Dataset and Sample Collection

This research included 832 individual ewe milk samples (50 mL), all of which were obtained during the morning milking and subsequently stored at 4 °C in sterile sealed containers until analysis. To analyze the influence of breed on the relationships between milk composition and coagulation, single-breed flocks were randomly selected: 8 Manchega flocks from the region of Castilla-La Mancha, 8 Merino de Grazalema flocks from the province of Cádiz, 8 Merino de Los Pedroches flocks from the province of Córdoba, and 8 Assaf flocks from the region of Castilla y León. Each flock was visited several times, and multiparous ewes with different lactation stage were randomly selected, resulting in a total of 26 ewes per flock and 208 milk samples per breed. The sample population comprised ewes with an average parity of 3.8 ± 1.9, an average prolificacy of 1.56 ± 0.56 lambs per lambing, and 79.4 ± 28.6 days in milk (DIM), with no significant differences between breeds (verified by ANOVA, with a level of significance of *p* < 0.05).

### 2.3. Laboratory Analysis

All analyses were performed upon receipt of the samples at the Dairy Laboratory (Universidad de Córdoba, Spain), on the same day as the sampling. The initial pH of the milk was determined using a Crison Bassic pH meter (Crison Instruments S.A., Barcelona, Spain), and the milk composition variables were measured on a Milkoscan FT-120 (Foss Electric, Hillerød, Denmark), including fat, crude protein, lactose, and total solids. The milk coagulation properties (MCP) were determined on a Formagraph viscometer (Foss Electric, Hillerød, Denmark) after the addition of 50 μL of a single-strength bovine rennet dilution (chimosyn-pepsine 80:20, 185 IMCU/mL) at 4% to 10 mL of milk, to reach 0.037 IMCU/mL of milk [40]. The MCP included rennet clotting time (RCT), curd firming time (k_20_), and curd firmness at 60 min (A_60_). The coagulation efficiency (A_60_/RCT) was also calculated [41]. Following coagulation in the Formagraph, the curds were cut with a spatula and drained by centrifugation (30 min at 2800× *g* and 37 °C), after which they were weighed to calculate individual laboratory curd yield (ILCY) [42], which was expressed in g/10 mL of milk.

### 2.4. Data Analysis

The preliminary testing of the data was carried out to determine outliers to be discarded before further analysis. Because the data had different measurement units, they were standardized to zero mean and a unit standard deviation. The common descriptive characteristics of the studied variables are shown in Table 1.

First, the bivariate association between variables was explored using Pearson correlations, avoiding variables with correlation coefficients exceeding an absolute value of >0.95 to prevent multicollinearity in the models. To examine the association between the breed and the composition and coagulation properties of the milk, ANOVA was employed, with the Student–Newman–Keuls (SNK) test used as a means contrast test.

Subsequently, a canonical correlation analysis (CCA) was used to examine the interrelations between milk composition and coagulation properties, and to explore whether these relationships are consistent across the four breeds or if there are breed-specific interrelations. This methodological approach has recently been employed to analyze similar interrelations in cow, sheep, and goat milk [43]. The technique was developed by Hotelling [44] and deals with the degree of linear association between two sets of variables, X and Y. The basic principle of CCA is the construction of pairs of canonical variables (U_i_, V_i_), which are linear combinations of the original variables, so that each pair is orthogonal to the previous one and represents the best explanation of the set *Y* (formed by *q* dependent variables) with respect to the set *X* (formed by *p* independent variables) that has not been obtained by the previous pairs. These linear combinations reflect the relationship between both sets of variables [45].

A CCA was deemed appropriate since it provides the magnitude of the relationships that may exist between two groups of variables, and a quantification of the relative contribution of each variable to those relationships. Five CCA models were carried out, one for each population breed and one for the entire data set. By comparatively evaluating the CCA models, it is possible to establish similarities and differences at the breed level [46].

All statistical analyses were performed using Statgraphics Centurion XVI (StatPoint Technologies Inc., Warrenton, VA, USA). The significance level was assumed as *p* < 0.05.

## 3. Results

A total number of 10 samples did not coagulate within 60 min under laboratory conditions and, thus, were not considered for this analysis. Therefore, the total sample size was 822 individual milk samples.

The results of the composition and coagulation obtained for each breed are shown in Table 2. In general terms, milk from Manchega and Merino de Grazalema were the richest in fat and protein. Manchega milk coagulated slowly compared to the other breeds, but led to firmer clots and to the obtention of an intermediate curd yield. The highest curd yield corresponded to Merino de Los Pedroches, with also a fairly slow coagulation but a firm clot. Milk from Assaf, on the other hand, presented the poorest composition, resulting in soft curds and the lowest curd yields of all four breeds. 

Table 3 shows Pearson correlations obtained between the analysed variables. The set of variables was highly correlated, although no pair of variables showed a correlation higher than 0.95. The highest level of correlation was found between fat and total solids content (r = 0.90), and fat again was the trait that was highly correlated more often to the others (4 out of 9 times with an absolute value over 0.40).

Five CCA models were built, the main characteristics of which are shown in Table 4. High and statistically significant canonical correlations were found between composition and milk coagulation properties for each breed separately and for the whole set of breeds. The relationships between both sets of variables were stronger in the breed Assaf, intermediate in the Merino breeds (Merino de Los Pedroches and Merino de Grazalema), and weaker in the breed Manchega. 

**Figure 1:**
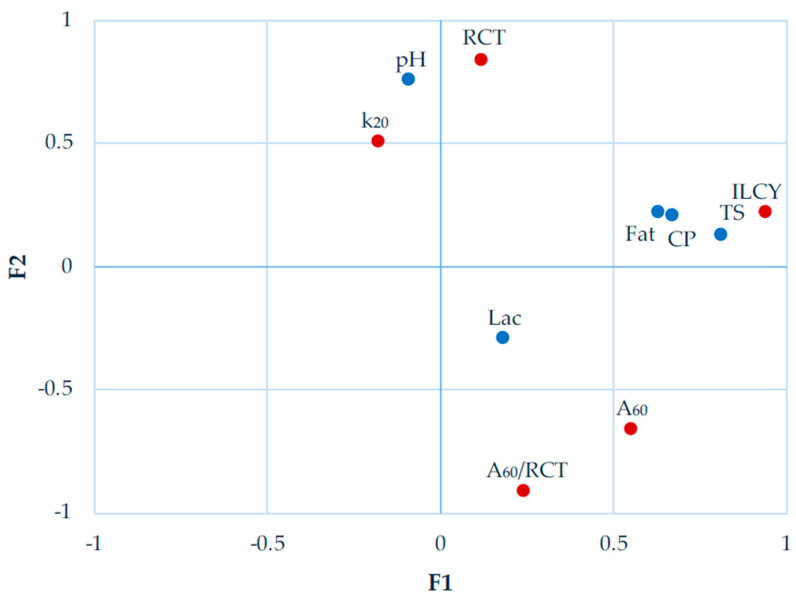
Canonical correlation analysis similarity map determined by the first and second canonical variates for milk composition traits (**●**) and milk coagulation properties (**●**) for the whole dataset.

Figure 1 shows the correlation structure between the analysed variables and the two first pairs of significant canonical components (F_1_ and F_2_) for the model established with the whole dataset.

Taking into account all the breeds together, the first four pairs of canonical variables (F_1_ to F_4_) were found significant (Table 4), although canonical correlations were considerably weak from F_3_ onwards, explaining less than 10% of variability. The first pair of canonical variables explained 66.8 % of variability and, with a correlation of 0.705, it links positively curd yield with total solids, fat and protein in milk. The second pair of canonical variables (F_2_) explained 24.6% of variability, had a correlation of 0.428 and relates the coagulation process with initial pH of milk, highlighting that milk alkalinisation tends to lead to slower coagulation, resulting in softer curds.

**Figure 2:**
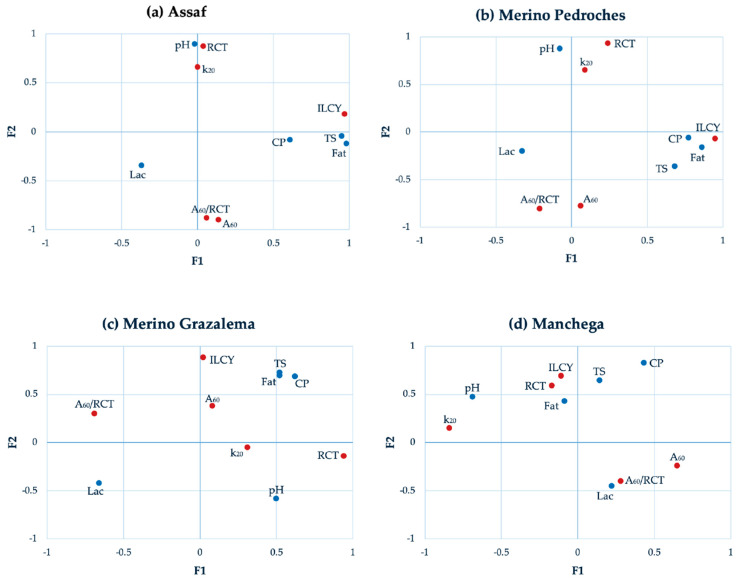
Canonical correlation analysis similarity map determined by the first and second canonical variates for milk composition traits (**●**) and milk coagulation properties (**●**) in each population: (**a**) Assaf; (**b**) Merino de Los Pedroches; (**c**) Merino de Grazalema; and (**d**) Manchega.

The models developed for the breeds Assaf and Merino de Los Pedroches, maintain the correlation structure previously described for the whole dataset (Figure 2). In both breeds, the main pattern of covariation (F_1_) is given by the positive relationship between curd yield and the fat and protein content of milk, while the relationship between pH and the coagulation process is weaker and shows a lesser percentage in variance (F_2_). However, the breeds Manchega and Merino de Grazalema showed their own patterns of variance, which differ from the general correlation structure (Figure 2). 

In Merino de Grazalema, the first pair of canonical components (F_1_) links milk composition to its coagulation process—instead of to curd yield—and reveals a tendency to coagulate faster when milk solids are richer in lactose and poorer in fat and protein, while the second pair of canonical components (F_2_) positively relates curd yield with milk fat and protein. 

In Manchega, the main pattern of covariation is established between pH and the milk coagulation process (F_1_) and shows, as in the rest of breeds, that higher pH values lead to the obtention of softer curds and slower coagulation. The second pair of canonical components relates milk nutrient content—specifically crude protein—with curd yield, showing that the latter tends to increase when coagulation is slower. However, Manchega is the only breed where this relationship can be observed (Figure 2). 

## 4. Discussion

In past decades, there has been a growing interest in the characterization of autochthonous breeds, as productions are strongly regionalized and contribute to the preservation of biodiversity and rural traditions, leading to environmental and economic sustainability [2,24]. This acquires great importance in the case of small dairy ruminants, since production not only involves important cultural and sociological aspects, but is also regarded by consumers as a source of high-quality products, addressing their concerns on the origin and nature of dairy foods [8,47]. 

This sector has traditionally been focused on the obtention of high milk yields and a good compositional quality to achieve optimal cheese production. So, many authors have outlined milk major components as largely responsible for good performance during this manufacturing process [15,23,48,49,50]. As for the present study, all milk samples analysed were within the normal ranges described for milk solids in ewe milk. The differences and similarities in milk composition and coagulation properties observed in this study align with findings from other previous studies [51,52,53]. It is worth highlighting that, for some breeds, RCT were found to be relatively long. References regarding the MCP of the four studied breeds in particular are scarce, but similar values have been described for Assaf [49,54] and the Merino breeds [38,55] when using the same rennet composition and concentration as in the present study. Manchega milk coagulated under these particular conditions shows a wider range of RCT values, while those obtained in this study are in line with previous work from our research group [56]. However, coagulation times are in general still longer than those reported by other authors for sheep milk, and this must certainly be investigated further. Although the rennet used in all cases has similar enzymatic composition and ratio, the long RCT values obtained in the present study are believed to be due to the lower rennet concentration used, compared to that described by others when analysing coagulation traits of sheep milk (0.037 vs. 0.0513 IMCU/mL of milk) [57,58].

In recent years, canonical correlation analysis has been used in some studies involving dairy ruminants for the analysis of aspects such as milk immunoglobulin profiles, genomic characterisation, or the impact of housing on animal health [59,60,61,62]. Moreover, a recent study from our research group has used CCA to explore species-specific differences in milk processing [43]. However, to our knowledge, there are no available studies that use this approach to analyse differences and similarities in the technological performance of milk from different breeds.

The results from the CCA revealed patterns of interrelation that are common to the four analyzed breeds, as well as specific interactions that distinguish them from each other. These differences may possibly be linked to the rusticity of native breeds, strongly adapted to the particular environments of each Spanish region, frequently harsh and adverse to cost-effective dairy production [63]. This has historically been the case with Manchega, the most important Spanish dairy sheep breed, but is also reflected in other autochthonous breeds such as Churra and Latxa [54]. Hence, the emergence of third-country breeds in the early 80s, as is the case of Assaf, introduced into dairy flocks (whether in purity or by crossbreeding) to enhance milk yields, led to more intensified production systems [18,35]. Differences in composition and coagulation can also be attributed to a wide variety of factors, including genetic and physiological characteristics of each breed, as well as their environment and management [43]. Each breed can have different genetic profiles, that may include variations in genes responsible for protein and fat synthesis in milk, as well as others related to enzymatic activity and casein structure, which strongly affect the milk coagulation capacity [64,65]. Additionally, production conditions, such as nutrition and feeding type, climate, environmental stress, flock management, and milking practices, can also play a significant role in the observed variations in composition and coagulation traits [11,24,66]. Furthermore, other factors such as herd health and the occurrence of disease can influence milk quality and technological capacity [14,67].

Nevertheless, milk coagulation is a complex phenomenon, and there is still debate about the factors that should be included as traits of interest in sheep and goat breeding schemes [20,68,69]. Currently, selection continues to focus almost exclusively on the obtention of high milk production or on maximizing curd yield. However, the latter is not directly pursued, but attempted through the selection of fat and protein as traits of interest [20], whose concentration paradoxically depends on milk yield, often leading to a dilution effect [70]. There is a positive association between ILCY and the milk nutrient content in all breeds, although this relationship varies in a particular manner according to breed. This is clearly reflected in the covariation patterns of Assaf and Merino de Los Pedroches, which were found to follow the general correlation structure obtained for the complete dataset. Specifically, in these two breeds, the relationship between ILCY and FAT, CP, and TS, is the most prominent among both sets of variables. Conversely, in Merino de Grazalema and Manchega, this relationship seems to be less relevant. In any case, previous research has evidenced that the technological performance does not only rely on composition, and milk with a high concentration of solids sometimes results in inefficient coagulation [41]. Thus, other possible approaches for optimizing cheese production involve the inclusion of MCP in breeding programmes. Studies focused on the genetic improvement of milk coagulation in dairy cows are fairly common, but there are few studies covering this topic on small ruminants [71]. Still, some authors have attempted to estimate the heritability of coagulation properties in native Spanish and Italian sheep breeds, which have been reported to be low or, in any case, moderate [17,72,73]. However, in certain cases, these estimated values suggest room for the genetic improvement of some technological traits (particularly coagulation time and curd firmness) which still need to be studied in depth.

Both aforementioned coagulation traits are highlighted in the correlation structure obtained in the present study for Manchega and Merino de Grazalema, linking MCP to lactose and pH rather than to the usual fat and protein contents, which seem to take a less important role in these two breeds. It is worth stressing the relevance of lactose and pH in the coagulation process. Lactose has often been regarded as the less important milk solid for cheesemaking, but recent studies consider this trait a biomarker of mastitis and, like pH, an indicator of udder health, since low lactose concentration is highly correlated to increased somatic cell counts (SCC) [19,70]. Moreover, milk from sheep with udder infections is also characterised by slightly alkaline pH values, which are known to compromise the stability of the casein micelles. This leads to impairments in the coagulation process, resulting in curds with a lower concentration of solids and a high moisture content, which can distort curd yield calculations [74]. This explains why in all the studied breeds except for Merino de Grazalema, more alkaline pH values were related to slower coagulation and the obtention of softer curds. In addition, for this particular breed, the results show that a lower lactose concentration seems to lead to slower coagulation. Many animal health strategies include lines of action to fight and control mastitis in dairy sheep breeds, and some authors propose the inclusion of lactose in selection programmes due to its correlation with other traits of interest [19,75]. In addition, recent research has explored genetic selection for mastitis resistance in dairy cows [76], but the results suggest that this is far from being successfully implemented in small ruminant species, due to, among other factors, its economic cost. Nevertheless, although the described parameters are considered good indicators of udder health, it would be useful to further explore other traits such as SCC and casein content, which should be included in future studies.

Some Spanish breeders’ associations such as AGRAMA (Manchega) and AMEGRA (Merino de Grazalema) currently maintain active lines of work focused both on animal health improvement and the technological control of milk intended for cheesemaking, and have a solid background of participation in research regarding the suitability of MCP as traits of interest for selection [46]. This might somehow explain why breeds that have, to some extent, considered MCP as traits of interest for genetic selection differ from the general pattern of covariation obtained for sheep milk. 

In summary, there are multiple pieces of scientific evidence that have marked a turning point in the debate between quantity or quality of milk and, for many of the aforementioned reasons, milk from native breeds has proven to have better coagulation [24,69]. Thus, the findings suggest that industry and dairy farmers should consider exploring new approaches for genetic selection in small ruminant breeding programmes, and explore strategies other than simply maximizing yields and milk solids. 

## 5. Conclusions

Milk quality relies on a wide range of husbandry systems and management strategies, often socio-culturally linked to the farming of different sheep breeds. In this context, canonical correlation analysis has proved to be a useful tool to explore relationships between traditional composition variables and the coagulation performance of milk. Although there is a common correlation structure for sheep milk coagulation, some breeds that have at some point considered including milk coagulation properties in breeding programs, show their own patterns of covariation that differ from the general trend in the cheese industry. This needs to be studied in depth, but outcomes from this perspective could lay the foundations for the industry to explore moving from the traditional approach of maximizing fat and protein to obtain higher curd yields, and consider other traits for selective breeding instead.

## Figures and Tables

**Table 1:** Description of milk composition and coagulation variables.

Variable	Description	Unit	Mean	S.E.
Milk composition (X-set)				
Fat	Fat content	%	6.63	0.05
CP	Crude protein	%	5.50	0.02
Lac	Lactose content	%	5.10	0.02
TS	Total solids	%	18.20	0.07
pH	pH	−log [H+]	6.70	0.00
Coagulation (Y-set)				
RCT	Rennet clotting time	min	28.97	0.26
k_20_	Curd firming time	min	4.54	0.17
A_60_	Curd firmness at 60 min	mm	49.07	0.38
ILCY	Curd yield for 10 mL of milk	g/10 mL	3.06	0.02
A_60_/RCT	Coagulation efficiency at 60 min	mm/min	1.94	0.02

**Table 2:** Results from ANOVA (mean ± SD) for milk composition and coagulation properties.

Variable	Assaf	Merino Los Pedroches	Merino Grazalema	Manchega
Fat	5.10 ± 1.41 ^c^	6.46 ± 1.02 ^b^	7.99 ± 1.65 ^a^	8.08 ± 1.38 ^a^
CP	5.22 ± 0.54 ^b^	5.67 ± 0.66 ^a^	5.65 ± 0.58 ^a^	5.68 ± 0.72 ^a^
Lac	5.24 ± 0.30 ^b^	5.93 ± 0.43 ^a^	4.59 ± 0.30 ^c^	4.54 ± 0.38 ^c^
TS	16.10 ± 1.70 ^c^	19.03 ± 1.86 ^b^	19.80 ± 2.20 ^a^	19.31 ± 1.61 ^b^
pH	6.68 ± 0.10	6.67 ± 0.10	6.68 ± 0.10	6.69 ± 0.11
RCT	27.64 ± 9.80 ^c^	31.66 ± 11.73 ^b^	25.30 ± 6.61 ^d^	35.38 ± 7.57 ^a^
k_20_	5.79 ± 10.33 ^a^	4.43 ± 1.85 ^b^	3.53 ± 2.15 ^b^	3.48 ± 0.98 ^b^
A_60_	42.41 ± 11.14 ^c^	59.03 ± 16.67 ^b^	43.31 ± 6.82 ^c^	62.54 ± 9.33 ^a^
ILCY	2.52 ± 0.41 ^c^	3.80 ± 0.53 ^a^	3.13 ± 0.77 ^b^	3.24 ± 0.52 ^b^
A_60_/RCT	1.81 ± 0.97 ^a^	2.36 ± 1.43 ^b^	1.84 ± 0.59 ^a^	1.89 ± 0.59 ^a^

^a–d^: Means without a common superscript are statistically different (*p* < 0.05). Fat = fat content; CP = crude protein; Lac = lactose; TS = total solids; pH = initial pH; RCT = rennet clotting time; k_20_ = curd firming time; A_60_ = curd firmness at 60 min; ILCY = individual laboratory curd yield; A_60_/RCT = coagulation efficiency.

**Table 3:** Pearson correlations between all the studied variables.

	Fat	CP	Lac	TS	pH	RCT	k_20_	A_60_	ILCY
Fat									
CP	0.53								
Lac	−0.57	−0.28							
TS	0.90	0.66	−0.36						
pH	−0.27	0.17	0.34	−0.21					
RCT	0.09	0.16	ns	0.08	0.28				
k_20_	0.09	0.16	ns	−0.11	0.23	0.40			
A_60_	0.19	0.22	0.13	0.28	−0.27	−0.28	−0.39		
ILCY	0.48	0.43	0.08	0.56	ns	0.18	ns	0.27	
A_60_/RCT	ns	ns	0.20	0.05	−0.28	−0.75	−0.32	0.64	ns

Fat = fat content; CP = crude protein; Lac = lactose; TS = total solids, pH = initial pH; RCT = rennet clotting time; k_20_ = curd firming time; A_60_ = curd firmness at 60 min; ILCY = individual laboratory curd yield; A_60_/RCT = coagulation efficiency. ns = not significant.

**Table 4:** Canonical analysis on milk composition (*X*) and milk coagulation properties (*Y*).

Population	Canonical Variates	Eigenvalue	Variance %	Canonical Correlation	Lambda	*p* Value
All breeds	F_1_	0.497	66.8	0.705	0.384	<0.001
	F_2_	0.183	24.6	0.428	0.764	<0.001
	F_3_	0.050	6.7	0.225	0.935	<0.001
	F_4_	0.014	1.9	0.120	0.985	<0.001
	F_5_	0.000	0.0	0.024	0.999	ns
Assaf	F_1_	0.653	60.9	0.808	0.214	<0.001
	F_2_	0.304	28.4	0.552	0.617	<0.001
	F_3_	0.089	8.3	0.297	0.887	<0.001
	F_4_	0.026	2.4	0.162	0.973	0.008
	F_5_	0.000	0.0	0.025	0.999	ns
Merino Los Pedroches	F_1_	0.423	58.5	0.650	0.414	<0.001
	F_2_	0.225	31.1	0.474	0.717	<0.001
	F_3_	0.063	8.7	0.250	0.925	0.011
	F_4_	0.009	1.2	0.096	0.987	ns
	F_5_	0.003	0.4	0.059	0.996	ns
Merino Grazalema	F_1_	0.412	62.5	0.642	0.500	<0.001
	F_2_	0.180	27.3	0.424	0.765	<0.001
	F_3_	0.047	7.1	0.218	0.933	0.021
	F_4_	0.015	2.3	0.124	0.979	ns
	F_5_	0.005	0.8	0.072	0.994	ns
Manchega	F_1_	0.268	51.7	0.518	0.557	<0.001
	F_2_	0.194	37.5	0.440	0.761	<0.001
	F_3_	0.047	9.1	0.217	0.944	ns
	F_4_	0.007	1.4	0.081	0.991	ns
	F_5_	0.002	0.4	0.045	0.998	ns

ns = not significant.

## Data Availability

The data presented in this study are available upon request from the corresponding author.
